# ESR Study of (La,Ba)MnO_3_/ZnO Nanostructure for Resistive Switching Device

**DOI:** 10.1186/s11671-017-1961-8

**Published:** 2017-03-09

**Authors:** Taras Polek, Mykhaylo Semen’ko, Tamio Endo, Yoshinobu Nakamura, Gurmeet Singh Lotey, Alexandr Tovstolytkin

**Affiliations:** 1 Institute of Magnetism, 36b Vernadsky Boulevard, Kyiv, 03680 Ukraine; 2 Department of Physics, Taras Shevchenko National University of Kyiv, Volodymyrska Street, 64/13, Kyiv, 01601 Ukraine; 3 Sagamihara Surface Lab, 1880-2 Kamimizo, Chuoku, Sagamihara, Kanagawa 252-0243 Japan; 4 University of Tokyo, 7 Chome-3-1 Hongo, Bunkyo, Tokyo, Japan; 5 Department of Physics, Nano Research Lab, DAV University, Jalandhar, Punjab 144012 India

**Keywords:** Substituted manganites, Oxide nanostructures, *p*–*n* heterojunctions, Rectification factor, Resistive switching, Electron spin resonance, Magnetic phase separation, 75.47.m, 73.40.Ei, 75.75.a, 76.50.g, 75.30.m

## Abstract

Structure, electric, and resonance properties of (La,Ba)MnO_3_/ZnO nanostructure grown on SrTiO_3_ (001) substrate have been investigated. It is found that at room temperature and relatively low voltages (|*V* |< 0.2 V), the structure shows good rectification behavior with rectification factor near 210. Resistive switching properties are detected after application of higher voltages. Temperature evolution of magnetic phase composition of the sample is analyzed in detail, based on results of electron spin resonance measurements. It is shown that magnetic state below 260 K is characterized by coexistence of ferromagnetic and paramagnetic phases, but no evidence of magnetic phase separation is revealed at higher temperatures. Different driving mechanisms for resistive switching, such as magnetic phase separation and/or electric field-induced migration of oxygen vacancies, are discussed in the context of obtained results.

## Background

Oxides of transition metals offer an attractive platform for new applications in electronics, spintronics, and optoelectronics. Complex oxides with perovskite structure occupy a special place among all oxide materials, since they display a rich variety of fascinating properties such as high-temperature superconductivity [1], colossal magnetoresistance (CMR) [2], left-handed properties [3, 4], and others. A peculiar feature of the CMR materials, to which belong manganese-based perovskites such as La_1-*x*_Me_*x*_MnO_3_ (Me = Ba, Sr, Ca…), is that paramagnetic to ferromagnetic transition is accompanied by a transition from *p*-type semiconducting to metallic conductivity [2, 5]. Due to a number of competitive interactions, the ground state of bulk and thin films of La_1-*x*_Me_*x*_MnO_3_ is often phase separated and usually characterized by a coexistence of highly conductive ferromagnetic (FM) phase and poorly conductive paramagnetic (PM) or charge-ordered phases [2]. Such complexity of the properties of perovskite manganites has become the point of attraction for a vast number of researchers around the world [2, 5, 6]. It is expected that this class of materials will create the foundation for various applications, such as low-field magnetoresistance sensors, resistive switching devices, and others [6, 7].

On the other hand, ZnO is a typical *n*-type semiconductor with a 3.4-eV band gap [8, 9]. This material is transparent for a visible spectrum but absorbs ultraviolet. There have been reports about ultraviolet stimulated emission and lasing from ZnO thin films [8]. The oxide is also promising for such applications as sensors and organic solar cells [9–11].

Thin films of oxides, such as ZnO, La_1-*x*_Ba_*x*_MnO_3_ (LBMO), La_1-*x*_Sr_*x*_MnO_3_ (LSMO), Nd-doped SrTiO_3_, and others, often display resistive switching properties, i.e., sharp change of resistivity under electric field or current, and are promising for the use in non-volatile memory elements [12]. To date, there has been no unique explanation of this phenomenon, although some models for the driving mechanisms of resistive switching have been proposed. The authors of [13] concluded that formation of a conducting path within a dielectric layer is responsible for the phenomenon in a metal/binary oxide/metal sandwich. They succeeded in direct observation of the inhomogeneous spatial structure of the conducting path, which was formed upon the initial voltage application. A thermal or electrochemical redox reaction in the vicinity of the interface between the oxide and the metal electrode was suggested as other plausible mechanism for resistive switching [14]. It was concluded, however, that further research from chemical, electronic, and crystallographic viewpoints is needed to elucidate actual microscopic mechanism because the chemical, electronic, and crystallographic properties of the oxides, as well as the metal electrodes, affect the mechanism.

A great deal of efforts has been concentrated on fabrication and investigation of oxide-based *p*–*n* heterojunctions. Zhang et al. [15] found that the *p*–*n* junction formed from ZnO nanosheet on LSMO thin film showed a high rectification factor of 120 at room temperature. Khachar et al. [16] reported magnetic field modulations of current–voltage (*I*–*V*) characteristics for ZnO/(La,Pr,Sr)MnO_3_/(Sr,Nb)TiO_3_-substrate system. The rectification factor was not very high, but the current was field sensitive at 300 K. The authors suggested that the modification of the rectifying behavior can be ascribed to the interface effects due to the change of the crystal field splitting of manganese *d* levels. The optical and magnetic field induced transformations of current–voltage characteristics of ZnO/LSMO nanostructure grown on LaAlO_3_ substrate were studied in Ref. [17]. It was suggested that the shape and size of the energy barrier were changed by the modifications of junction interface and interface tensile strain due to optical and magnetic external perturbations.

The attempts to tune resistive, rectifying, and magnetic properties of zinc oxide/substituted manganite nanostructures have been made by the authors of Refs. [17–21], who, in particular, studied the effects of temperature and magnetic field [18], changes in thickness of ZnO [14] and manganite [19] layers, effects of substrate kind and white light irradiation [20], etc.

Okada et al. [22] fabricated and investigated ZnO/substituted manganite bilayers on different substrates. They reported rectification properties and resistive switching over a wide temperature range. It was suggested that the driving mechanism for resistive switching is a coexistence of highly conductive FM phase and poorly conducting charge-ordered or PM phase in manganite layer. Taking into account that the existence of mixed magnetic/conductive state is a characteristic feature of substituted manganites [23, 24], further investigations are needed to examine the magnetic state of such structures and, thus, prove or disprove the above suggestion. One of the most efficient methods suitable for this aim is electron spin resonance (ESR), which makes it possible to separately analyze the parameters of different coexisting magnetic phases. This work is aimed at investigation of ESR properties of LBMO/ZnO nanostructure grown on SrTiO_3_ (STO) substrate and elucidation of the driving mechanism for the resistive switching properties.

## Methods

The LBMO/ZnO nanostructure was fabricated on SrTiO_3_ (001) substrate by ion-beam sputtering from (La,Ba)MnO_3_ and ZnO targets, as described in Ref. [22]. The deposition was performed with ultralow deposition rate (near 10^−3^ nm/s) in two stages: at first, ZnO underlayer was grown on SrTiO_3_ (001) substrate at 400 °C, and then secondary LBMO overlayer was deposited on the ZnO underlayer at 600 °C. The layer thickness, estimated as a product of deposition time and deposition rate, was around 100 nm for each of two layers.

The sample was characterized by the X-ray DRON-4 diffractometer using Co*К*α radiation. X-ray data were separately collected from both the bilayer and substrate sides.

Current–voltage characteristics were measured using a standard four-point probe method. Indium electrodes were placed on the surface of LBMO/ZnO bilayer having the step-shape, two electrodes on the surface of each layer. Ohmic behavior of the contacts was proved based on the observation of linear current–voltage characteristics for each pair of electrodes (placed either on LBMO or on ZnO layer).

ESR studies were carried out in the temperature range 100–300 K with the use of ELEXSYS E500 spectrometer equipped with an automatic goniometer. The operating frequency was *f* = 9.47 GHz. The temperature dependences of the ESR spectra were studied in two geometry configurations: external magnetic field is perpendicular and parallel to the film plane (*θ* = 0^o^ and 90°, respectively, where *θ* is an angle between external magnetic field **H** and film normal). In addition, the out-of-plane angle dependences of the resonance fields, *H*_res_(*θ, T* = const), were investigated at selected temperatures.

## Results and Discussion

**Fig. 1:**
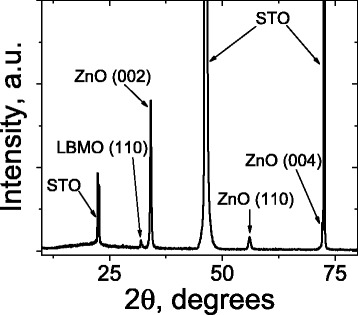
X-ray diffraction patterns for the LBMO/ZnO nanostructure

X-ray diffraction patterns, obtained by the collection of the data from the substrate side, contain only STO (001), (002), (003), and (004) reflections. The lattice parameter determined from the positions of (003) and (004) reflections is *a* = 0.39069(18) nm, which is close to the data of Ref. [25] (*a* = 0.39050 nm). The diffractogram, taken from the bilayer side, shows LBMO (110) peak and ZnO (002), (110), and (004) peaks in addition to the STO substrate peaks (Fig. 1). The main orientation of ZnO is (001), and LBMO has single phase of (110).

The LBMO/ZnO nanostructure shows fine rectification behavior at room temperature (295 K), and for relatively low voltage (|*V* |< 0.2 V), the *I*–*V* characteristics are fully reversible. Under these conditions, rectification factor is near 210.

**Fig. 2:**
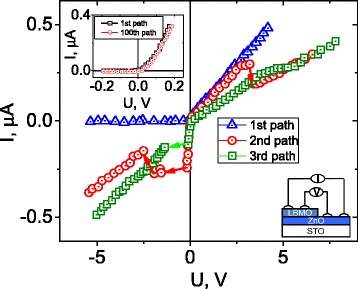
Current–voltage characteristics of the LBMO/ZnO nanostructure during the 1st, 2nd, and 3rd measurement cycles. *Inset* compares the first and hundredth low-voltage *I*–*V* characteristics, obtained in a series of separate experiments with |*V* |< 0.2 V

To prove the reversibility of the low-voltage *I*–*V* characteristics, 4 sets of measurements at regular intervals (4 days) were carried out. Each set included 25 measurement cycles with |*V* |< 0.2 V. Inset of Fig. 2 compares the first and hundredth *I*–*V* characteristics. It is seen that the difference is negligible.

Quite different behavior is observed after application of higher voltages. Figure 2 shows evolution of current–voltage characteristics of the LBMO/ZnO sample as the number of *I*–*V* measurement cycles increases. The duration of one measurement cycle was 2 min. Each subsequent measurement cycle was carried out in 10 min after the previous one. The original *I*–*V* curve is characteristic of a typical *p*–*n* junction. However, after running the first measurement cycle, the *I*–*V* dependence shows a clear one-step switching near 3 V in the forward and a clear two-step switching around −0.2 and −2.0 V in the reverse bias. In addition, it shows a very low resistance near zero Volts in the reverse side. Thus, the sample shows the transition from the very low resistant state, to the intermediate resistant state, and finally to the high resistant state with increasing the reverse voltage [22]. Further, after running the second measurement cycle, the *I*–*V* dependence shows a kink near 4 V in the forward (disappearance of one-step switching) and the one-step switching in the reverse bias (disappearance of two-step switching). On the whole, the forward current decreases while the reverse current increases with increasing the number of measurement cycles. This implies that the change of electric state is not caused by sample degradation. It is noteworthy that the original rectification behavior is usually resumed after a few days, indicating that high-voltage-induced state is metastable.

To study the temperature evolution of the LBMO magnetic phase composition, ESR measurements were carried out in the temperature range from 120 to 300 K. Two geometric configurations were chosen for measurements: external magnetic field **H** was either perpendicular (⊥) or parallel (||) to the film plane. Such measurement procedure makes it possible to separate the signals originated from FM and other magnetic phases, if they are present in the sample [26].

**Fig. 3:**
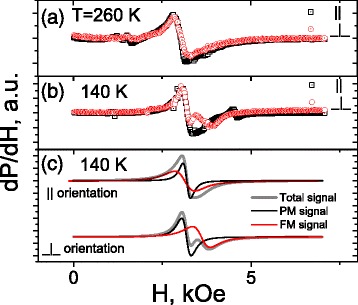
Representative ESR spectra for parallel (||) and perpendicular (⊥) orientations of the LBMO/ZnO plane with respect to external magnetic field **H**. **a**, **b** Experimental data at 260 and 140 K, respectively. **c** The results of the decomposition of the total signal into PM and FM components. Each component is described by the field derivative of Lorentzian

Representative ESR spectra for 260 and 140 K are shown in Fig. 3. At 260 K, the spectrum consists of one resonance signal whose parameters (position on the field scale and linewidth) are insensitive to the sample orientation (within the measurement error). The resonance field (*H*_res_ ≈ 3120 Oe) corresponds to Lande *g*-factor approximately equal 2, which is a characteristic of PM state of substituted manganites [23]. At the same time, at 140 K, more complicated behavior is observed. Comparison of the spectra for perpendicular and parallel measurement geometries makes it possible to conclude that the spectrum consists of two resonance signals. The first one with resonance field *H*_res1_ ≈ 3230 Oe is the signal from PM phase. Its position on the field scale does not depend on the sample orientation. The position of the second signal is sensitive to the orientation of the sample with respect to the external magnetic field. For perpendicular orientation, this signal has resonance field ${H}_{\mathrm{res}2}^{\perp }$ ≈ 3690 Oe, and it is well discernible in the total spectrum. Such behavior is a characteristic of the signal originated from FM phase [23, 27].

Very narrow weak signals, observed in all spectra under discussion, originate from paramagnetic impurities in the substrate [24], and they are not analyzed in this work.

Magnetic resonance in FM state is governed by the effective magnetization, and this depends on the spontaneous magnetization as well as anisotropies due to shape, strain, and crystallinity. For isotropic thin films, in which we assume anisotropy only due to shape, the resonance may be described using the Kittel’s equations with appropriate demagnetizing factors [27]:

1 $$ {\left(\omega /\gamma \right)}^2={H}_{\mathrm{res}}^{\left|\right|}\left({H}_{\mathrm{res}}^{\left|\right|}+4\pi {M}_0\right) $$

2 $$ \omega /\gamma ={H}_{\mathrm{res}}^{\perp }-4\pi {M}_0 $$

where ${H}_{\mathrm{res}}^{\left|\right|}$ and ${H}_{\mathrm{res}}^{\perp }$ are, respectively, the resonance fields for H parallel and perpendicular to the film plane, ω is angular frequency, and γ is gyromagnetic ratio.

**Fig. 4:**
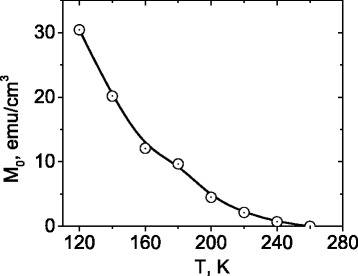
Temperature dependence of effective magnetization *M*_0_ calculated from Kittel’s equations

Figure 4 shows temperature dependence of the effective magnetization *M*_0_ obtained by the solution of the system of Eqs. (1)–(2) with the use of experimental resonance fields ${H}_{\mathrm{res}}^{\left|\right|}$ and ${H}_{\mathrm{res}}^{\perp }$. It is seen that *M*_0_ decreases from 30 emu/cm^3^ to zero as temperature rises from 120 to 260 K. At temperatures higher than 260 K, the sample is in PM state.

It should be noted that bulk LBMO displays FM to PM transition near 340 K [2, 5]. The reduced Curie temperature in the LBMO/ZnO nanostructure may be caused by relatively strong lattice mismatch between LBMO and ZnO [22].

**Fig. 5:**
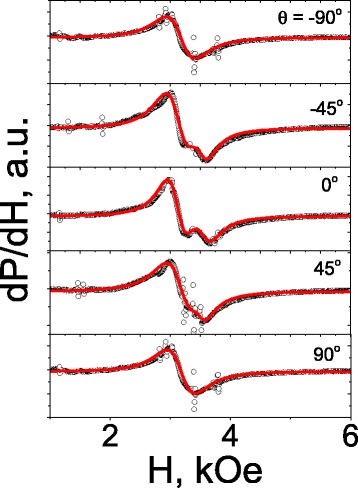
Out-of-plane angle dependences of the ESR spectra for the LBMO/ZnO nanostructure at 160 K. *Symbols* denote experimental data; *solid lines* are the results of the fitting of spectra by formula (3)

Taking into account the complexity of the ESR spectra and non-negligible background noise, additional measurements were made to prove the reliability of the obtained results. Detailed angle dependences of the ESR spectra may provide additional important information about magnetic state of a complex system. Such studies were carried out for the LBMO/ZnO nanostructure at *T* = 160 K. Figure 5 shows the spectra obtained for different values of the angle *θ* between external magnetic field **H** and film normal. Here again, we see relatively intensive absorption line whose resonance field does not depend on *θ* (signal from PM phase) and the line of weaker intensity, whose position changes as the sample is rotated (signal from FM phase).

The spectra were analyzed by fitting to the sum of the derivatives of Lorentzians [28]:

3 $$ \frac{d P}{ d H}\infty \frac{d}{ d H}\left\{\frac{\Delta {H}_1}{{\left( H-{H}_{\mathrm{res}1}\right)}^2+\Delta {H}_1^2}\right\}+\frac{d}{ d H}\left\{\frac{\Delta {H}_2}{{\left( H-{H}_{\mathrm{res}2}\right)}^2+\Delta {H}_2^2}\right\} $$

where *H*_res*i*_ and Δ*H*_*i*_ (*i* = 1,2) are, respectively, resonance field and width of the corresponding line. The results of the fitting are shown in Fig. 5 by solid lines.

**Fig. 6:**
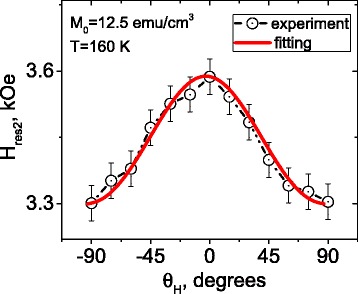
*H*_res2_ vs *θ*_*H*_ dependence for the LBMO/ZnO nanostructure (*T* = 160 K). *Symbols* denote experimental data; *solid lines* are the results of the fitting of experimental data with the use of Eqs. (5)–(6)

Figure 6 shows the angle dependence of the resonance field *H*_res2_ for FM phase (circles). To analyze the *H*_res2_ vs *θ* dependence, one should go beyond the Kittel’s equations. In general, the resonance conditions for a FM system are governed by the behavior of the density of free energy *E* and can be found from the equation [29]:

4 $$ {\left(\frac{\omega}{\gamma}\right)}^2=\frac{1}{{\left({M}_0 \sin {\theta}_M\right)}^2}\left({E}_{\theta_M{\theta}_M}{E}_{\phi_M{\phi}_M}-{E}_{\theta_M{\phi}_M}^2\right) $$

where *θ*_*M*_ and *ϕ*_*M*_ are, respectively, polar and azimuthal coordinates of effective magnetization (**M** = (*M*_0_, *θ*_*M*_, *ϕ*_*M*_)), and *E*_*ij*_ (*i*,*j* = *θ*_*M*_ *, ϕ*_*M*_) denote partial derivatives of *E* with respect to *θ*_*M*_ and *ϕ*_*M*_ variables. In the case of a thin FM film, the energy density is [30]:

5 $$ E=-{M}_0 H\left[ \sin {\theta}_H \sin {\theta}_M \cos \left({\phi}_H-{\phi}_M\right)+ \cos \left({\theta}_H-{\theta}_M\right)\right]+2\pi {M}_0^2 $$

where *θ*_*H*_ and *ϕ*_*H*_ are, respectively, polar and azimuthal coordinates of external magnetic field: **H**  = (*H*, *θ*_*H*_, *ϕ*_*H*_).

An additional necessary condition for the resonance to be fulfilled is the minimization of the energy of FM sample with respect to the angles *θ*_*M*_ and *ϕ*_*M*_:

6 $$ \partial E/\partial {\theta}_M = 0,\kern1em \partial E/\partial {\phi}_M = 0 $$

In our case, since the measurements were carried out for *ϕ*_*M*_ = const, only polar angles are remained in Eqs. (5)–(6).

After numerical solution of the system of Eqs. (5)–(6), the *H*_res2_ vs *θ*_*H*_ dependence was obtained, which is shown in Fig. 6 by solid line. Satisfactory agreement between the experimental and fitted data makes it possible to calculate the value of effective magnetization: *M*_0_ = 12.5 emu/cm^3^. This value is close to that obtained earlier from Kittel’s equations *M*_0 Kittel_ ≈ 12.1 emu/cm^3^.

It was suggested in works [21, 22, 31] that the driving mechanism for resistive switching in the LBMO/ZnO nanostructures is a coexistence of highly conductive FM phase and poorly conducting charge-ordered phase in manganite layer. Our investigations confirm the existence of mixed magnetic state at temperatures lower than 260 K. At the same time, they show that at temperatures higher than 260 K, the manganite layer is paramagnetic. This, however, does not ultimately exclude the magnetic phase separation as possible driving mechanism for resistive switching at temperatures exceeding 260 K. There is a possibility of the existence of tiny amount of FM phase above 260 K which cannot be detected by the present ESR analysis. This phase could form very thin percolating paths which could be easily disconnected by the current heating, causing the switching from lower to higher resistance state.

Other possible driving mechanism for resistive switching, namely temperature-enhanced electric-field-induced migration of oxygen vacancies, was suggested in Ref. [32] for nanoscale manganite structures. The authors of this work use combination of electric fields and Joule self-heating to change the oxygen stoichiometry and promote oxygen vacancy drift in a free-standing La_0.7_Sr_0.3_MnO_3_ thin film microbridge placed in controlled atmosphere. By controlling the local oxygen vacancies concentration, the LSMO-based microbridges were reversibly switched from metallic to insulating behavior on timescales lower than 1 s and with small applied voltages (<5 V). Multiple resistive states were shown to be set by selected current pulses which determined different oxygen vacancy profiles within the device.

## Conclusions

Structure, electric, and resonance properties of LBMO/ZnO nanostructure fabricated on SrTiO_3_ (001) substrate by ion-beam sputtering have been studied in this work. The layer thickness is near 100 nm for each of two layers. X-ray diffraction studies show that the main orientation of ZnO is (001), and LBMO has single phase of (110).

The LBMO/ZnO nanostructure shows fine rectification behavior at room temperature (295 K), and for relatively low voltage (|*V* |< 0.2 V), the *I*–*V* characteristics are fully reversible. At the same time, resistive switching is observed after application of higher voltages.

ESR measurements have been carried out in the temperature range from 120 to 300 K. It is demonstrated that nucleation of ferromagnetic phase occurs at 260 K and magnetic state below this temperature is characterized by coexistence of ferromagnetic and paramagnetic phases. As temperature decreases below 260 K, the effective magnetization of ferromagnetic phase increases and reaches 30 emu/cm^3^ at 120 K. No evidence of magnetic phase separation is found that at temperatures higher than 260 K.

Possible driving mechanisms for resistive switching have been discussed. It is concluded that magnetic phase separation and/or electric field-induced migration of oxygen vacancies, may be operative in the sample under investigation.
